# Intimate Partner Violence and the HIV Care and Treatment Cascade Among Adolescent Girls and Young Women in DREAMS, South Africa

**DOI:** 10.1097/QAI.0000000000002843

**Published:** 2021-10-29

**Authors:** Andrew Gibbs, Tarylee Reddy, Kalysha Closson, Cherie Cawood, David Khanyile, Abigail Hatcher

**Affiliations:** a Gender and Health Research Unit, South African Medical Research Council, Pretoria, South Africa;; b Centre for Rural Health, School of Nursing and Public Health, University of KwaZulu-Natal, Durban, South Africa;; c Biostatistics Research Unit, South African Medical Research Council, Durban, South Africa;; d School of Population and Public Health, The University of British Columbia, Vancouver, British Columbia, Canada;; e Epicentre, Durban, South Africa;; f University of North Carolina, Chapel Hill, NC; and; g University of the Witwatersrand, Johannesburg, South Africa.

**Keywords:** violence, adherence, treatment, women, gender, South Africa

## Abstract

**Background:**

Intimate partner violence (IPV) may affect the HIV-treatment cascade.

**Setting:**

Four high HIV-prevalence DREAMS health districts in South Africa.

**Methods:**

Secondary analysis of cross-sectional data collected March 2017–June 2018, using random household sample of young (12–24 years) girls and women. Face-to-face interviews assessed IPV and HIV-status knowledge, and finger-prick blood draws assessed ART (antiretroviral therapy) uptake and viral suppression. We used logistic regression to estimate crude and adjusted effects of IPV on HIV knowledge, ART uptake, and viral suppression.

**Results:**

Of 18,230 adolescent girls and young women, 8413 (46%) reported ever having had sex, of whom 1118 (13%) were HIV positive. The 90:90:90 benchmarks were 61% knew their status, 86% had ART present in their blood sample, and 91% were virally suppressed. Among the entire sample of young women living with HIV, 65.6% were virally suppressed. Past year IPV was reported by 15%. In adjusted models, IPV trended toward increasing the odds that a young woman was aware she was living with HIV [adjusted odds ratios (aOR) = 1.40, 2.00–9.98, *P* = 0.067]. There was no association between IPV and reduced treatment use (aOR = 0.73, 0.41–1.29). IPV was independently associated with reduced viral suppression (aOR = 0.30, 0.13–0.66).

**Conclusions:**

Addressing the role of IPV in undermining the treatment cascade for adolescent and young women is a critical issue for HIV programming.

## Abstract 2

Supplemental Digital Content is Available in the Text.

## INTRODUCTION

The global goals of achieving “90:90:90 by 2020” aimed to ensure that by the end of 2020 90% of people living with HIV would know their HIV serostatus, 90% of those aware of serostatus would be on treatment, and 90% of those on treatment would be virally suppressed.^1^ This target recognized the benefit of treatment access and adherence for individuals living with HIV, as well as its impact on onwards transmission of HIV. This target, now passed, has expanded to 95:95:95 by 2030.

There was, however, substantial variation in progress toward 90:90:90.^1^ In South Africa, the country with the greatest number of PLHIV globally (∼7 million), although there have been important strides toward achieving 90:90:90, recent population representative data showed that only 84.9% of those living with HIV know their status, 70.6% of those tested are on treatment, and 87.5% of those treated were virally suppressed.^2^ Women, in particular adolescent girls and young women (aged 15–24), experience marked challenges with HIV testing, access and adherence to treatment, and achieving viral suppression.^3,4^ In a recent systematic review of studies across sub-Saharan Africa, 49% of young women aged 15–24 years achieved viral suppression (well below the 73% goal consistent with 90:90:90).^5^

One key driver of poor engagement in the cascade of HIV care and treatment for women is exposure to intimate partner violence (IPV).^6^ Studies suggest among women who experience violence HIV-testing maybe reduced because of fear of disclosure if HIV positive,^7,8^ although others suggest testing may increase as women realize they may be more at risk of acquiring HIV.^9^ A systematic review found women who experienced IPV were less adherent and had poorer engagement in HIV care and lower odds of viral suppression.^6^ A range of potential pathways from IPV to reduced engagement in care, adherence, and suppression have been described, including ongoing fear of HIV disclosure leading to hiding medication^7,10^ and greater challenges in attending medical appointments because of less control over money and mobility.^7,10^ The systematic review, however, only drew on literature from high-income settings in North America and included no studies among younger female patients. More recent work from sub-Saharan Africa has demonstrated similar impacts of IPV on the HIV-treatment cascade,^7,11,12^ but without a focus on young women, nor population-based samples. This gap in the extant literature has limited our understanding of the impact of IPV on the 90:90:90 cascade, particularly among adolescent girls and young women in low-income and middle-income settings.

## METHODS

We aimed to fill the research gap on IPV and the HIV treatment cascade among young women through a secondary analysis of population-based data focused on adolescents and young women in marginalized communities in South Africa. We hypothesize that among sexually active women (aged 12–24), IPV is associated with reduced achievement of the 90:90:90 treatment targets, specifically:

1. Among those who are living with HIV, those who have experienced IPV have lower odds of being aware of their HIV-positive status;
2. Among those who know they are living with HIV, those who have experienced IPV have lower odds of current ART (antiretroviral therapy) use;
3. Among those who are currently accessing ART, those who experience IPV have lower odds of achieving viral suppression (<1000 copies/mL).

We conducted secondary analysis of cross-sectional, population-based data collected in 4 health districts from 2 provinces of South Africa: City of Johannesburg and Ekurhuleni (in Gauteng Province), and eThekwini and uMgungundlovu (in KwaZulu-Natal) between February 2017 and July 2018.

Details of this study have been published elsewhere.^13^ In brief, in each health district, communities identified by government as high HIV-prevalence settings were targeted by the PEPFAR/USAID-funded program called DREAMS (which stands for “Determined, Resilient, Empowered, AIDS-free, Mentored, and Safe”).^13^ Within these settings, local NGOs selected communities to implement the DREAMS program. Using a multistage-stratified cluster-based sampling design, 4 health districts were the primary strata. The communities identified by the NGOs were mapped onto the census small areas layer, and a PPS (proportional to size, where size was total households) sample was drawn. Within each selected small areas layer, a random sample of 55 households were drawn and eligible household had an adolescent girl or young woman (aged 12–24) living in them.^13^

Trained fieldworkers identified selected households and screened residents to identify if there was an adolescent girl or young woman (aged 12–24) resident. If there were multiple young women, all were eligible. Further inclusion criteria were ability to provide informed consent (those older than 18) and for those younger 18 assent and parental/guardian consent. Those potential participants with cognitive challenges were ineligible. Female, trained interviewers conducted face-to-face interviews in locations with audio privacy in English, isiZulu, Sotho, and Afrikaans languages. Interviewers captured data onto preprogrammed tablets, with in-built logic and skip patterns, and the mean length of interviews was 45 minutes. Participants received a gift valued ∼US$3 for participation. Data were collected from May 2017 to June 2018.

The sample size was calculated for the primary analysis of the study, an estimate of HIV-incidence reduction in this population (based on assumption of 2 waves). Specifically a 40% reduction in the HIV-incidence rate, at the 5% significance level, was stratified by Province Gauteng and KwaZulu-Natal. These produced sample sizes of 10,500 in Gauteng and 8000 in KwaZulu-Natal.^13^

### Measures

#### Exposure of Interest

To assess recent IPV, we used behaviorally-specific measures of physical IPV (5 items) and sexual IPV (3 items), based on the WHO Violence Against Women Scale,^14^ revised for use in South Africa. An example item was: “In the last 12 months, how many times has a current or previous boyfriend or partner slapped you or thrown something at you which could hurt you?”, with responses “never”, “once,” or “more than once.” A response of “once” or “more than once” to any item was classified as having experienced IPV in the last 12 months. A response of “never” to every item was classified as nonexposed. These items were only asked of women who self-reported ever having had sex.

### Outcomes of Interest

To estimate the 90:90:90 cascade, we collected the following data. HIV prevalence was assessed by collecting 2 microcontainers of whole blood drawn from finger pricks. HIV status was assessed using HIV polymerase chain reaction test.^13^ All samples were tested with Genscreen Biorad HIV ½ Combi Assay, and positive results were confirmed by Western blot.

We asked all participants whether they knew their current HIV status (yes, no, or refuse to answer). Participants were offered to receive their HIV status at either a local health clinic within 2 weeks, or immediately using a rapid HIV-test, and if this indicated an HIV-positive diagnosis, they were referred to their local clinic for additional tests.^13^

To assess ART exposure among participants living with HIV, we tested the same plasma samples using high-performance liquid chromatography coupled with tandem mass spectrometry (Agilent HPLC-Module 1260 Infinity; Mass spectrometer- ABSciex 6.5+). Tests covered all regimens in use in the public health sector in these provinces. The screening was developed and validated in-house,^13^ with positivity based on a low cut-off level for each sample. Internal standards and negative controls were used to test batches.

Finally, all HIV-positive samples were also assessed for HIV-1 RNA viral load using Abbott M2000 Real Time PRC platform. The test provided values of >1000 copies/mL as a detectable viral load and 1000 copies/mL for undetectable viral load (ie, viral suppression) in the context of ART exposure.^13^

To estimate the first 90, the proportion of those living with HIV who know their status, we divided the number of those self-reporting being HIV-positive and/or ART exposure in bloods, by the total number who were living with HIV. We did this as some reported being HIV negative, but were positive for ART in their blood sample and this followed the main protocol.^13^ To estimate the second 90 (proportion of those knowing their HIV status on treatment), we divided the number testing positive for ART by those who self-reported being HIV positive and/or tested positive for ART. To calculate the third 90, we calculated the proportion virally suppressed by those on treatment.

Sociodemographic controls included current age, highest education level (less than primary, primary only, secondary only, or tertiary), and whether the women had ever been pregnant (yes/no). We asked 3 items about the past month household food insecurity using the Household Hunger Scale,^15^ summing items (range 0–9 Cronbach α = 0.93) with larger scores indicating greater food security, and treated as a continuous score. Additional covariates were selected based on prior research showing they were associated with the HIV-treatment cascade. Alcohol use was assessed as a binary where participants were asked how frequently in the past year they had drunk alcohol (never, monthly or less, 2–4 times a month, 2–3 times a week, or 4 or more times a week) and recorded as never or ever for analysis. Depressive symptoms were assessed using 5 items of the Center for Epidemiological Studies—Depression (CESD scale) (range 0–15, study Cronbach α = 0.86).^16^ The scale was summed, and higher scores indicated more depressive symptoms. Both alcohol use and depression have been shown to impact on the HIV-treatment cascade for women^17,18^ and may impact through making testing less likely, women forgetting medication, or making treatment adherence more challenging.

### Statistical Analysis

We described the sample of adolescents and young women who ever reported having had sex, providing percentages, means, and 95 percent confidence intervals (95% CIs). We then assessed descriptive associations between these and women's recent experience of IPV, with *t*-tests for continuous variables and χ^2^ tests for categorical variables. Finally, we estimated the unadjusted and adjusted associations between IPV and each 90:90:90 target using logistic regression. For adjusted models, we selected variables for inclusion in models that were associated with the outcome of interest and which displayed a 10% or greater change in the model coefficient (log odds) for the main effect (past-year IPV) in bivariate analyses. We present odd ratios, adjusted odds ratios (aORs), 95% CI, and *P*-values. There was no adjustment for missing data. All statistical analyses accounted for the stratified structure of the data, using the svy commands in Stata 16.

We ran a series of sensitivity analyses. We first modeled the main analysis with a range of covariates in the model (age, age-squared, and age, education, and food insecurity). We then reran the models separately assessing whether there was an independent association between physical IPV, and sexual IPV, and the treatment cascade. Finally, we did a subgroup analysis by age (12–19 and 20–24 years).

#### Ethics

The study was provided with ethical approval by the Biomedical Research Ethics Committee at the University of KwaZulu-Natal. All those 18 or older provided written informed consent for the study and for the blood samples. Those younger than 18 provided assent and a parent or guardian provided consent.

## RESULTS

In total, we recruited 18,230 adolescent girls and young women, of which 8413 (46.2%) reported ever having had sex. The mean age in the sexually active sample was 20 years (Table 1), three-quarters (76.0%) reported some secondary education and almost 1 in 5 (17.9%) reported some tertiary education. Among the sample, just over half (56.3%) reported ever having been pregnant and a third (32.9%) reported any alcohol use in the past year (Table 1).

**TABLE 1.** Sociodemographic and Health Outcomes Among Adolescent Girls and Young Women Reporting Ever Having Had Sex

	N	Description of Sample	Past Year IPV Experience—No	Past Year IPV Experience—Yes	*P*
		n (%)/Mean (95% CI)	n (%)/mean (95% CI)	n (%)/mean (95% CI)	
Age	8161	20.00 (19.94 to 20.07)	19.93 (19.85 to 20.00)	20.46 (20.30 to 20.60)	<0.001
Education	7826				0.003
None		229 (2.2%)	195 (3.1%)	34 (3.6%)	
Primary		242 (2.9%)	204 (2.8%)	38 (3.3%)	
Secondary		6023 (76.0%)	5160 (75.3%)	863 (79.5%)	
Tertiary		1332 (17.9%)	1193 (18.65%)	139 13.55%)	
Food insecurity (>=more food secure)	6596	7.8 (7.1 to 7.9)	7.9 (7.8 to 8.0)	7.2 (7.0 to 7.4)	<0.001
Health outcomes/risk factors					
Ever pregnant*	6186	3496 (56.3%)	2871 (54.6%)	625 (65.1%)	<0.001
Alcohol use in the last 12 months (yes)	8160	2517 (32.9%)	1941 (29.5%)	576 (52.3%)	<0.001
Alcohol use frequency (never)	8160	5664 (69.2%)	5086 (70.5%)	558 (47.7%)	<0.001
Alcohol use frequency (monthly or less)		1773 (21.7%)	1418 (21.6%)	355 (32.5%)	
Alcohol use frequency (2–4 times a mo)		539 (6.6%)	397 (6.0%)	142 (13.0%)	
Alcohol use frequency (2–3 times a wk)		151 (1.9%)	95 (1.5%)	56 (4.8%)	
Alcohol use frequency (4+ times a wk)		53 (0.7%)	30 (0.4%)	23 (1.9%)	
Depressive symptoms (>=more)	8161	1.47 (1.29 to 1.55)	1.30 (1.22 to 1.37)	2.47 (2.28 to 2.67)	<0.001
HIV positive (yes)†	8135	1118 (13.2%)	903 (12.3%)	215 (18.2%)	<0.001
Phys/sex IPV past year (yes)	8161	1134 (14.8%)			
90:90:90 cascade					
Know their status, if HIV positive	1118	697 (61.2%)	555 (60.1%)	142 (65.5%)	0.171
Of those know their status, proportion on treatment	696	602 (85.8%)	487 (87.1%)	115 (80.9%)	0.086
Of those on treatment, proportion virally suppressed	602	552 (91.5%)	455 (93.3%)	97 (84.3%)	0.003

* Thirty refused to answer (included in denominator).

† Determined via the polymerase chain reaction test. To assess associations, for categorical variables, Pearson's χ^2^ tests, and for continuous tests, bivariate linear regression. All accounted for the structure of the data set.

Of those who had ever been sexually active, 13.2% (n = 1118) were HIV positive as indicated by polymerase chain reaction testing. Among those who were HIV positive, 61.2% knew their status (first 90). Among those who knew their HIV-positive status, 85.8% had ART present in their blood sample (second 90). Of those with any ART exposure, 91.4% were virally suppressed (last 90). Among the entire sample of HIV-positive young women, 65.6% were virally suppressed.

Past year physical and/or sexual IPV was 14.8%. A total of 1005 young women (13.9%) reported any physical IPV, and a total of 342 young women (4.5%) reported any sexual IPV in the last 12 months.

Descriptively (Table 1), those reporting past year IPV had a higher mean age and a lower proportion reported secondary education. The mean scores for food security were lower among those reporting past year IPV (indicating greater food insecurity). A greater proportion of young women reporting IPV had ever been pregnant, mean scores for alcohol use were higher, and mean depressive symptoms scores were higher. A higher proportion of those reporting IPV were also HIV positive.

**FIGURE 1.**
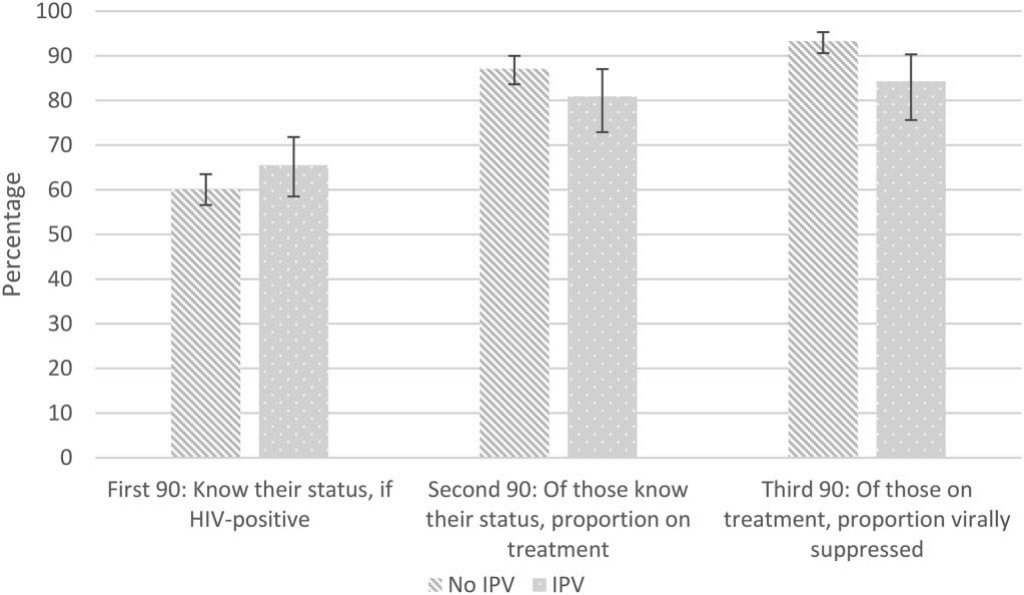
90:90:90 cascade by IPV experience.

For the first 90, awareness of HIV status, there were no differences (Fig. 1) descriptively between those who experienced IPV and those who did not. For the second 90, ART access, a lower proportion among those who experienced IPV were on ART (80.9% vs. 87.5%) but this was not significantly different to those who did not experience IPV. For the third 90, viral suppression, a significantly lower proportion of those experiencing IPV had viral loads <1000 copies/mL (84.3% vs. 93.3%).

In crude and adjusted models (Table 2), IPV trended toward increasing the odds that a young woman was aware of her HIV-positive status (aOR = 1.40, 0.98–2.00, *P* = 0.067). In addition, IPV was independently associated with a lower odds of viral suppression in crude and adjusted models (aOR = 0.37, 0.18–0.75). Full models are available in Table 1, Supplemental Digital Content, http://links.lww.com/QAI/B749.

**TABLE 2.** Unadjusted and Adjusted Associations Assessing Between IPV Experience and the 90:90:90 Cascade

	OR (95% CI)	*P*	aOR (95% CI)	*P*
Know their status if HIV positive*	1.26 (0.90 to 1.75)	0.172	1.40 (0.98 to 2.00)	0.067
Of those who know their status on treatment†	0.63 (0.36 to 1.07)	0.088	0.73 (0.41 to 1.29)	0.28
Of those on treatment, virally suppressed‡	0.39 (0.20 to 0.74)	0.004	0.37 (0.18 to 0.75)	0.006

* Sample size: n = 1118 unadjusted and n = 996 adjusted for age, education, alcohol use, depression, and food security.

† Sample size: n = 696 unadjusted and n = 663 adjusted, for age, alcohol use, and food security.

‡ Sample size: n = 602 unadjusted and n = 569 adjusted, for age, food security, and ever pregnant.

Sensitivity analyses confirmed the primary findings, for just sociodemographic covariates (see Table 2, Supplemental Digital Content, http://links.lww.com/QAI/B749), and models with just physical IPV experience and just sexual IPV experience (see Table 3, Supplemental Digital Content, http://links.lww.com/QAI/B749).

For subgroup analysis by age, we found in the younger group (12–19 years—see Table 4, Supplemental Digital Content, http://links.lww.com/QAI/B749) similar patterns and magnitude of effect sizes seen to the full sample, but 95% CIs passed through zero. For those 20–24 years (see Table 5, Supplemental Digital Content, http://links.lww.com/QAI/B749), there remained a significant association between IPV experience and reduced viral suppression (aOR 0.27, 0.11–0.71), whereas the direction of association between knowledge of HIV status and IPV experience was reversed, although not significant. We also assessed whether the cascade varied by ever being pregnant but found no differences.

## DISCUSSION

The 90:90:90 targets have successfully reached a large portion of adolescent girls and young women in South Africa. However, gaps remain, and these may be related in part to IPV experience. In the last 12 months, 14% of young women reported experience of physical IPV and 5% sexual IPV. IPV experience was associated with 70% lower odds of viral suppression among those living with HIV and on treatment in adjusted analysis.

For the first 90 (knowledge of HIV status), a lack of association between status awareness and IPV may be due to generally high HIV testing rates in South Africa among women, primarily during pregnancy. Over half the women reported ever being pregnant, and HIV testing is routine in the antenatal setting.^19–21^ It is also plausible that testing outreach efforts in South Africa adequately engaged young women who are also exposed to IPV in relationships. Finally, a young woman who is exposed to IPV may be aware that she is at risk of sexually transmitted infections^22^ or may have more engagement with the health system due to injuries or other health sequelae of violence. With this backdrop in mind, it is not surprising that IPV trends toward increasing rates of HIV status awareness.

In contrast to previous research,^11,12,23^ although aligned with other findings,^19,23–25^ there was no clear indication that experience of IPV reduced access to treatment among those who knew their HIV-positive status. While there was a 7 percentage point reduction in ART use among those experiencing IPV, and a marginal (*P* < 0.1) association in crude models, no association persisted after adjusting for potential confounders.

The association between IPV experience and reduced odds of viral suppression supports previous research from high-income countries^19–21^ and 2 studies in Zambia and South Africa.^26,27^ However, it contrasts with much literature from low-income settings. A study among 129 South African adolescents identified no association between IPV exposure and viral loads,^28^ and of 357 female sex workers in Kenya, those who reported IPV had better viral suppression than nonvictimized counterparts.^29^ The distinct finding in our data may result from the population-based nature of the survey, because participants were not active in clinical programs, and is certainly also a factor of the sample sizes required to detect an association.

That the association between IPV and viral suppression remained after adjusting for theoretically important confounders is an advance for the field. Although we cannot tease apart mechanisms for this association, several plausible pathways are noted. Violence may have a biological impact on HIV progression as IPV has a range of biological impacts such as inflammation and dysregulation.^30–32^ It is also possible that IPV exposure hindered adherence to ART after it was initiated, as other research has demonstrated.^20,24,33^ In qualitative research, adult women describe hiding their medicine, avoiding taking treatment at strategic moments to stay safe, and being barred by violent partners from visiting the clinic to obtain treatment.^34^ Nonadherence may be partially mediated by depression, which is predicted by IPV exposure^35^ and, in turn, predicts poor adherence.^36,37^

The age-stratified analysis showed no significant differences among those aged 12–19 and those 20–24 compared with the primary analysis. The sustained association in the younger cohort is important as it suggests addressing the interlinked association between IPV and HIV-treatment outcomes needs to begin early and confirms previous research on the impact of violence on adolescent girls' treatment outcomes.^26^

Determining whether a causal association exists between IPV and objective biomarkers like viral suppression is important for the HIV field. Previous research in Canada highlighted the impact of “severe violence” among women living with HIV as associated with increased mortality,^38^ suggesting the effects of IPV on women's health may marked. To intervene strategically, we require longitudinal analyses and better insights into the biological and social mechanisms through which IPV may impact on HIV-related health and engagement in care.

For young women living with, or at risk of acquiring, HIV, whose sexual relationship dynamics are often influx, it will be crucial to assess patterns of relationship safety over time. This moment in the life course can be treated as a “window of opportunity” for the health system to skillfully intervene. However, 2 recent systematic reviews highlight that there are virtually no published HIV engagement in care interventions tailored to the needs of adolescent women.^39,40^ Our findings suggest that testing may not be the most impactful place to target IPV care and support (striking given its emphasis in the literature^41,42^) but rather it may be best focused on sustaining ART engagement. Peer support groups,^43^ tech-assisted chat platforms,^44^ clinic referrals for safety and counseling, and broader social protections^45^ may be promising strategies to assist young women in addressing IPV within the context of HIV care and treatment. Each of these–or combinations of these—require urgent attention by researchers and programmers.

The study has limitations. It was cross-sectional, limiting our ability to assess causality. Associations are better studied prospectively because of the episodic nature of IPV and potentially cyclical pattern of engagement in HIV care and treatment. While the sample was population-based and representative of the communities DREAMS works in, it is not generalizable to all adolescent girls and young women in South Africa. We assessed IPV in the past year, whereas self-reported HIV status, ARVs in the blood, and viral suppression were assessed at the point of data collection, which may attenuate the association between IPV and outcomes. The prevalence of IPV was lower than seen in other studies due to either population-based sampling or to respondent bias through face-to-face interviews, which may decrease willingness to report IPV.^46^ Because we were focused on IPV, we only assessed IPV among those reporting ever having sex, although IPV can occur in nonsexual relationships, and other forms of violence (eg, from caregivers) are associated with worse adherence among children and adolescents.^47^ We did not assess emotional IPV, which is the most prevalent form of IPV, and associated with HIV-treatment outcomes. Our analysis assumed anyone who had biologically confirmed ARVs in their blood knew they were living with HIV; although given the longevity of ARVs, it may have included some people who were not currently taking ARVs. The test for viral suppression could only be used to dichotomize at 1000 copies, and so, we may have missed some people who were not virally suppressed. There was also missing data, although it is unclear how this would have biased the results. We did not undertake multiple imputations because in each step, less than 10% of data were missing. The sample was population-based, although only generalizable to DREAMS communities in the 4 health districts. Finally, we did not assess whether lack of viral suppression among those on ART was driven by emerging ART resistance, although how this would impact on the association is unclear.

## CONCLUSIONS

In this study, adolescent girls and young women in a population-based sample in South Africa achieved viral suppression at a rate lower than 90:90:90 targets. Experience of recent IPV was associated with lower odds of achieving viral suppression (the third 90), although other steps in the cascade were not significantly different among IPV-exposed young women. The need to address gender inequalities and specifically IPV has been a central premise of HIV programming and research for many years, often led by women living with HIV themselves.^10^ If the directionality of IPV impacting on viral suppression remains in longitudinal analyses, addressing the role of IPV in undermining the treatment cascade for adolescents is a critical issue for HIV programming—and may have monumental knock-on effects for other priority areas such as adolescent mental health, pregnancy prevention, and schooling attainment. Designing and implementing effective IPV interventions is critical for improving health adolescent girls and young women living with HIV.

## Supplementary Material

SUPPLEMENTARY MATERIAL
