# Supplementary material for: Intimate Partner Violence and the HIV Care and Treatment Cascade Among Adolescent Girls and Young Women in DREAMS, South Africa
Source: J Acquir Immune Defic Syndr. 2021 Oct 29;89(2):136–42. doi: 10.1097/QAI.0000000000002843 (PMC8740602; doi:10.1097/QAI.0000000000002843)
Supplement: SUPPLEMENTARY MATERIAL [file qai-89-136-s001.docx]

Supplementary Tables

| **Supplementary Table 1: adjusted associations assessing between IPV experience and the 90:90:90 cascade** | | | | | | | |
| --- | --- | --- | --- | --- | --- | --- | --- |
|  | Know their status if HIV-positive^a^ | | Of those know their status on treatment^b^ | | Of those on treatment, virally suppressed^c^ | |  |
|  | aOR(95%CI) | pvalue | aOR(95%CI) | pvalue | aOR(95%CI) | pvalue |  |
| Past year IPV | 1.40(0.98, 2.02) | 0.067 | 0.71(0.40, 1.25) | 0.234 | 0.37(0.19, 0.75) | 0.006 |  |
| Age | 1.12(1.04, 1.21) | 0.004 | 0.93(0.82, 1.06) | 0.267 | 1.31(1.09, 1.58) | 0.005 |  |
| Education None | ref |  | xx |  | xx |  |  |
| Primary only | 1.45(0.48, 4.42) | 0.51 | xx |  | xx |  |  |
| Secondary only | 0.79(0.42, 1.51) | 0.48 | xx |  | xx |  |  |
| Tertiary | 0.45(0.22, 0.93) | 0.032 | xx |  | xx |  |  |
| Alcohol use past year | 0.55(0.40, 0.75) | <0.001 | 0.84(0.51, 1.39) | 0.5 | xx |  |  |
| Depressive scores (>=more) | 1.03(0.98, 1.08) | 0.296 | xx |  | xx |  |  |
| Food insecurity (>=more) | 0.99(0.93, 1.05) | 0.712 | 1.13(1.04, 1.24) | 0.007 | 1.13(0.99, 1.27) | 0.061 |  |
| Ever pregnant | xx |  | xx |  | 1.58(0.77, 3.26) | 0.214 |  |
|  | ^a^ Sample size: n=1118 unadjusted & n=996 adjusted for age, education, alcohol use, depression and food security; ^b^ Sample size: n=696 unadjusted & n= 663 adjusted, for age, alcohol use, food security; ^c^ Sample size: n=602 unadjusted & n=569 adjusted, for age, food security and ever pregnant; xx= not included in the final models | | | | | |  |
|  |  |  |  |  |  |  |  |
|  |  |  |  |  |  |  |  |

| **Supplementary Table 2: Adjusted associations between IPV and the 90:90:90 cascade, adjusting for a range of socio-demographics** | | | | | | |
| --- | --- | --- | --- | --- | --- | --- |
|  | **Know their status if HIV-positive** | | | | | |
|  | aOR(95%CI)^a^ | pvalue | aOR(95%CI)^a^ | pvalue | aOR(95%CI)^b^ | pvalue |
| Past year IPV | 1.25(0.90, 1.75) | 0.186 | 1.25(0.90, 1.74) | 0.188 | 1.23(0.86, 1.76) | 0.256 |
| Age | 1.11(1.04, 1.17) | 0.001 |  |  | 1.22(1.04, 1.21) | 0.003 |
| Age - squared |  |  | 1.00(1.00, 1.00) | 0.001 |  |  |
| None |  |  |  |  | ref |  |
| Primary only |  |  |  |  | 1.40(0.45, 4.31) | 0.559 |
| Secondary only |  |  |  |  | 0.86(0.46, 1.62) | 0.636 |
| Tertiary |  |  |  |  | 0.47(0.23, 0.96) | 0.039 |
| Food insecurity (>=more) |  |  |  |  | 0.99(0.94, 1.05) | 0.809 |
|  | ^a^ Sample size n=1116, ^b^ Sample size n=974 | | | | | |
|  | **Of those know their status on treatment** | | | | | |
|  | aOR(95%CI)^c^ | pvalue | aOR(95%CI)^c^ | pvalue | aOR(95%CI)^d^ | pvalue |
| Past year IPV | 0.62(0.36, 1.06) | 0.082 | 0.62(0.36, 1.06) | 0.82 | 0.70(0.38, 1.29) | 0.257 |
| Age | 0.92(0.83, 1.03) | 0.133 |  |  | 0.93(0.81, 1.06) | 0.288 |
| Age - squared |  |  | 1.00(1.00, 1.00) | 0.143 |  |  |
| None |  |  |  |  | ref |  |
| Primary only |  |  |  |  | 0.46(0.08, 2.77) | 0.398 |
| Secondary only |  |  |  |  | 0.88(0.25, 3.07) | 0.845 |
| Tertiary |  |  |  |  | 0.59(0.14, 2.42) | 0.459 |
| Food insecurity (>=more) |  |  |  |  | 1.15(1.04, 1.27) | 0.005 |
|  | ^c^ Sample size n=695; ^d^ Sample size n=616 | | | | | |
|  | **Of those on treatment, virally suppressed** | | | | | |
|  | aOR(95%CI)^e^ | pvalue | aOR(95%CI)^e^ | pvalue | aOR(95%CI)^f^ | pvalue |
| Past year IPV | 0.36(0.18, 0.69) | 0.002 | 0.35(0.18, 0.69) | 0.002 | 0.42(0.20, 0.88) | 0.021 |
| Age | 1.32(1.17, 1.49) | <0.001 |  |  | 1.45(1.19, 1.75) | <0.001 |
| Age - squared |  |  | 1.01(1.00, 1.01) | <0.001 |  |  |
| None |  |  |  |  | ref |  |
| Primary only |  |  |  |  | 1.40(0.09, 23.5) | 0.813 |
| Secondary only |  |  |  |  | 1.23(0.36, 4.16) | 0.743 |
| Tertiary |  |  |  |  | 1 |  |
| Food insecurity (>=more) |  |  |  |  | 1.17(1.03, 1.33) | 0.016 |
|  | ^e^ Sample size n=602; ^f^ Sample size n=509 | | | | | |

| **Supplementary Table 3: Adjusted associations assessing between physical IPV experience, sexual IPV, and the 90:90:90 cascade** | | | | |
| --- | --- | --- | --- | --- |
|  | Physical IPV | | Sexual IPV | |
|  | aOR(95%CI) | pvalue | aOR(95%CI) | pvalue |
| Know their status if HIV-positive | 1.43(1.00, 2.03)^a^ | 0.048 | 1.02(0.62, 1.68)^d^ | 0.924 |
| Of those know their status on treatment | 0.62(0.35, 1.24)^b^ | 0.106 | 0.93(0.37, 1.25)^e^ | 0.885 |
| Of those on treatment, virally suppressed | 0.43(0.21, 0.92)^c^ | 0.03 | 0.23(0.08, 0.71)^f^ | 0.011 |
| ^a^ Sample size: n=1116, adjusted for alcohol use; ^b^ Sample size: n=662 adjusted for food security; ^c^ Sample size: n=571 adjusted, for age, food security and alcohol use; ^d^ Sample size: n=1035 adjusted for education, alcohol use; ^e^ Sample size: n=662, adjusted for food insecurity; ^f^ Sample size: 509, adjusted for age, education, food insecurity | | | | |

| **Supplementary Table 4: Unadjusted and adjusted associations between IPV and 90:90:90 cascade for adolescent girls 12-19** | | | | |
| --- | --- | --- | --- | --- |
|  |  |  |  |  |
|  | Unadjusted and adjusted associations with IPV | | |  |
|  | OR(95%CI) | p-value | aOR(95%CI) ^d^ | pvalue |
| Know their status if HIV-positive^a^ | 1.05 (0.56,1.99) | 0.871 | 0.92 (0.45,1.87) | 0.809 |
| Of those know their status on treatment ^b^ | 0.71 (0.20, 2.61) | 0.609 | 0.60 (0.15,2.50) | 0.483 |
| Of those on treatment, virally suppressed ^c^ | 0.70 (0.23,2.16) | 0.536 | 0.30 (0.07,1.26) | 0.1 |
| ^a^ Sample size: n=254 unadjusted & n=192 adjusted, for age, education, alcohol use, ever pregnant; ^b^ Sample size: n=143 unadjusted & n= 114 adjusted, for food security, alcohol use and ever pregnant; ^c^ Sample size: n=128 unadjusted & n=100 adjusted for alcohol use and ever pregnant | | | | |

| **Supplementary Table 5: Unadjusted and adjusted associations between IPV and 90:90:90 cascade for adolescent girls 20-24** | | | | |
| --- | --- | --- | --- | --- |
|  | OR(95%CI) | p-value | aOR(95%CI) ^d^ | pvalue |
| Know their status if HIV-positive^a^ | 1.33 (0.91,1.94) | 0.142 | 1.47 (0.99,2.16) | 0.054 |
| Of those know their status on treatment ^b^ | 0.61 (0.34,1.09) | 0.098 | 0.76 (0.40,1.44) | 0.399 |
| Of those on treatment, virally suppressed ^c^ | 0.28 (0.13, 0.61) | 0.001 | 0.24 (0.10, 0.55) | 0.001 |
| ^a^ Sample size: n=864 unadjusted & n=864 adjusted for alcohol use and depressive symptoms; ^b^ Sample size: n=553 unadjusted & n= 553 adjusted for alcohol use, depressive symptoms and food security; ^c^ Sample size: n=474 unadjusted & n=442 adjusted for food security and ever pregnant | | | | |
